# Thrombotic Storm: A Case of Multiple Concurrent Thrombosis Secondary to Cocaine Use

**DOI:** 10.7759/cureus.59164

**Published:** 2024-04-27

**Authors:** Ramayee Nadarajan, Muhammad Areeb Ashfaq, Hajira Z Malik, Siva Parcha

**Affiliations:** 1 Internal Medicine, University of South Alabama, Mobile, USA; 2 Cardiology, University of South Alabama, Mobile, USA

**Keywords:** retroperitoneal hematoma, anticoagulation, myocardial infarction, left ventricular thrombus, mesenteric ischemia

## Abstract

Cocaine abuse is known to have deleterious effects on multiple organ systems. Its effects on the cardiovascular system are well-established in the literature. The presence of a left ventricular thrombus (LVT) is a well-recognized complication of an anterior myocardial infarction, especially in patients with aneurysmal formation. There is a paucity of reports where cocaine use and LVT are associated with myocardial infarction and mesenteric ischemia simultaneously. Our patient is a 49-year-old female, who presented to our institution after ingesting a large volume of cocaine. She complained of abdominal pain, chest pain, and was eventually found to have a left ventricular mural thrombus with concomitant superior mesenteric artery ischemia, and renal and splenic infarcts. Administration of therapeutic anticoagulation resulted in the development of retroperitoneal hematoma resulting in a therapeutic dilemma.

## Introduction

The relationship between cocaine ingestion and an acute coronary event is well-established in literature and is commonly encountered in clinical practice. The most affected organ systems by cocaine are the cardiovascular, nervous, respiratory, and renal systems. One of the mechanisms of injury is oxidative stress, causing mitochondrial damage and impacting several organ systems may be affected at the same time [1]. Cocaine use also induces a prothrombotic state in the body, as described below. There are no recently reported cases in the literature where cocaine use and a mural thrombus were present in a patient found to have concurrent ischemia in the coronary and mesenteric vascular systems. However, there are certain cases with simultaneous acute ischemic events in a patient with recent cocaine use [2,3]. We present one such case of a 49-year-old patient with a left ventricular thrombus and infarcts in the coronary and mesenteric circulation.

## Case presentation

A 49-year-old female presented to our facility for a 5-day history of chest pain characteristic of an acute coronary event, and abdominal pain worse after meals and associated with non-bloody diarrhea. She endorsed cocaine use in the days prior to the onset of symptoms and during the days she experienced the pain. She had a myocardial infarction (MI) 4 years prior to presentation but was subsequently lost to follow-up and had discontinued her antiplatelet therapy. Her vitals at presentation showed a pulse of 101 beats per minute, a respiratory rate of 21 breaths per minute, and a blood pressure of 158/117 mmHg. Physical examination was significant for a patient in discomfort, tachycardic with a regular rhythm, and no murmur or gallop. The abdomen was diffusely tender to palpation without rebound tenderness or guarding. 

**Figure 1:**
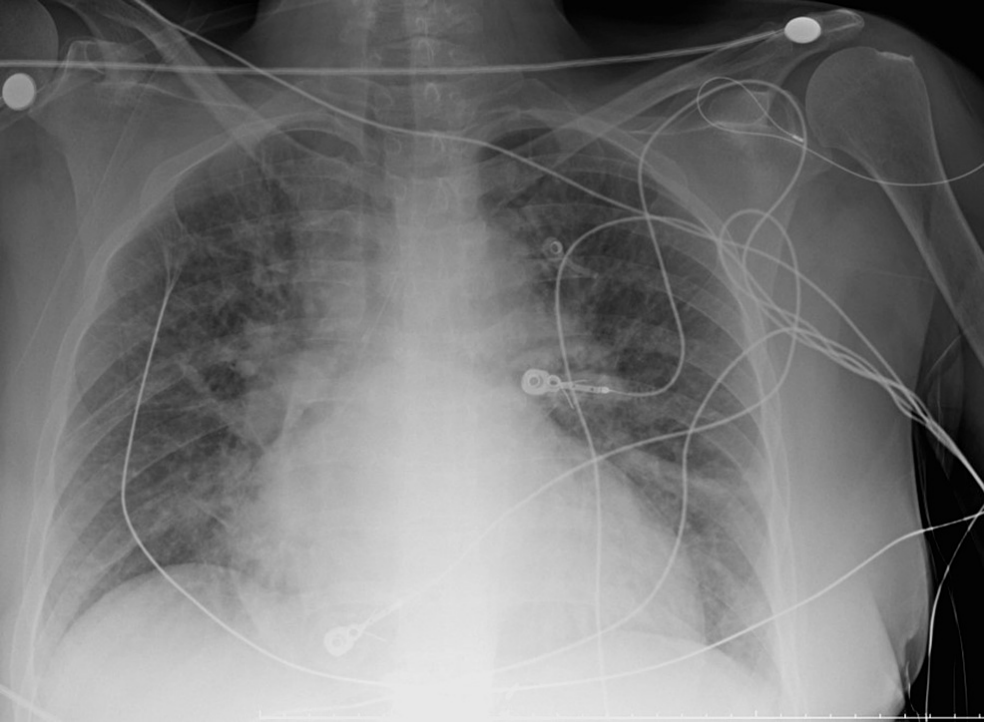
Chest x-ray showing pulmonary edema.

**Figure 2:**
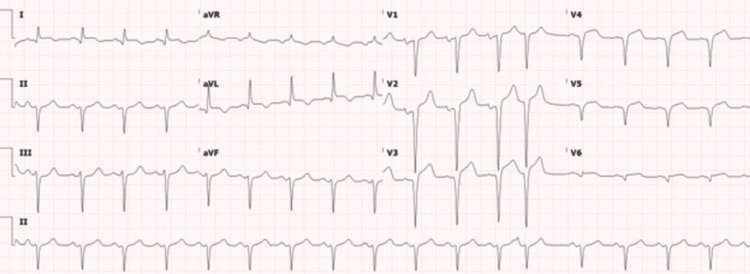
EKG showing sinus tachycardia with left axis deviation, and left atrial enlargement with an age-indeterminant anteroseptal infarct. EKG: Electrocardiogram.

The pertinent laboratory tests were as below (Table 1). Chest x-ray was significant for pulmonary edema (Figure 1). A 12-lead electrocardiogram showed sinus tachycardia and extensive Q waves with ST elevation with no reciprocal depression (Figure 2).

**Table 1:** Pertinent laboratory values on admission. NT-pro BNP: N-terminal pro b-type natriuretic peptide; pg/mL: picograms/millilitre; x10^3^/mcL: 1000 cells/microlitre; mmol/L: millimoles per litre.

	Reference Ranges	Results
Troponin	3 – 59 pg/mL	2387 pg/ml
NT-pro BNP	5 - 125 pg/mL	20169 pg/mL
White Cell Count	4.3 – 10.0 x10^3^/mcL	17.72 x 10^3^/mcL
Lactate	0.4 – 2.0 mmol/L	7.3 mmol/L

**Figure 3:**
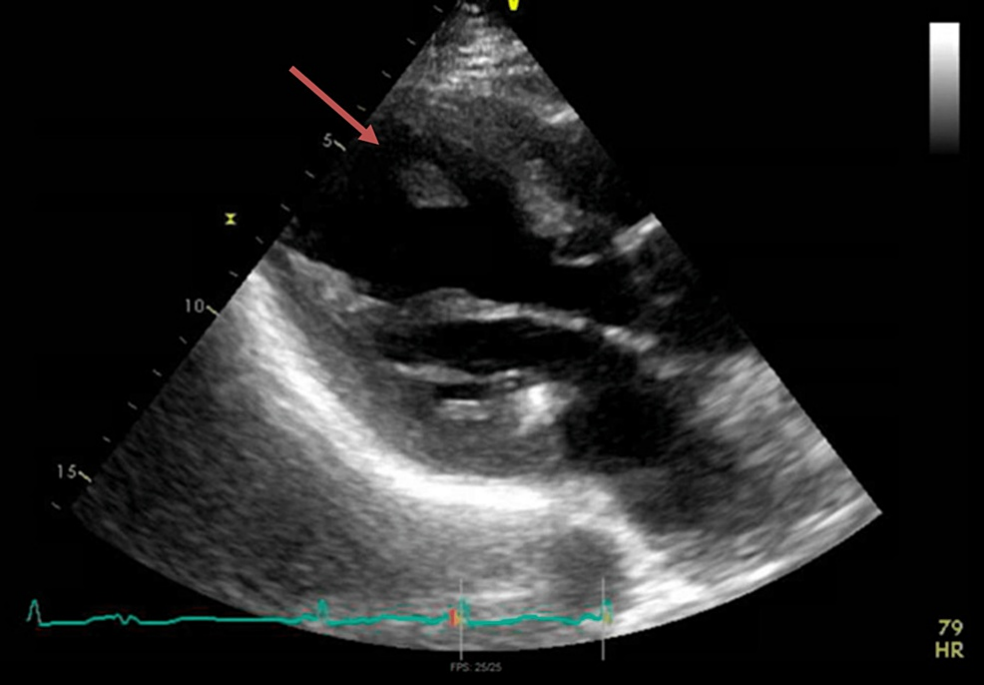
The left ventricle with a thrombus attached by a thin fibrinous string as shown by the red arrow on the echocardiogram parasternal long axis.

**Figure 4:**
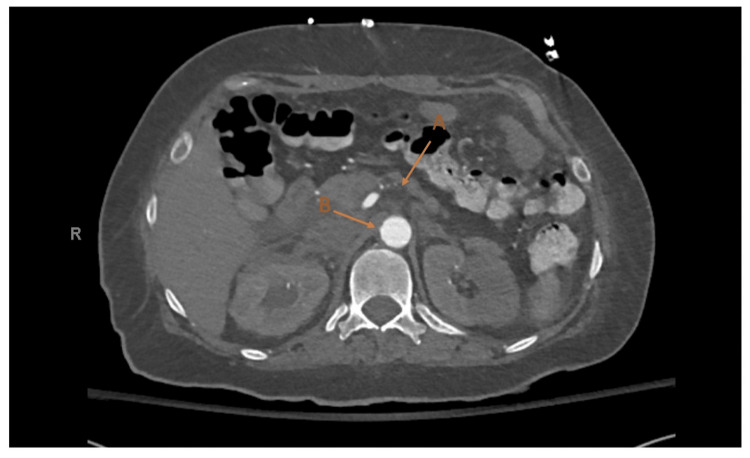
SMA occlusion with extension into the abdominal aorta on CT abdomen and pelvis with contrast A: SMA occlusion with lack of contrast. B: Aorta with contrast as comparison. SMA: Superior Mesenteric Artery; CT: Computerized Tomography.

The patient underwent an urgent transthoracic echocardiogram (TTE) which was significant for an ejection fraction less than 20% with diffuse global hypokinesis. There was also an apical multilobed echo density of 1.12 x 5 x 2.5 cm in size, consistent with a thrombus (Figure 3). The patient was taken for an urgent left heart catheterization, performed via right radial artery access, where she was found to have complete occlusion of the left anterior descending artery with no visible thrombus, and severe atherosclerotic coronary artery disease in the rest of the vessels. No immediate surgical intervention was possible for the complete occlusions. She was therefore medically managed with IV nitroglycerin which temporarily relieved her symptoms. This led us to believe she had episodes of coronary vasospasm. The patient's acute coronary syndrome was medically managed but there was concern regarding initiating antiplatelet therapy and heparin drip because the stool occult blood test resulted positive. A computed tomographic angiography (CTA) of the aorta with intravenous contrast showed mid-superior mesenteric artery (SMA) occlusion with dilated small bowel with mucosal thickening and no intestinal pneumatosis (Figure 4). The surgical team was consulted, and she underwent catheter-directed mechanical thrombectomy of the SMA. However, her symptoms did not resolve, and a repeat CTA demonstrated continued occlusion. She then underwent an exploratory laparotomy with open thrombectomy of the SMA and was postoperatively placed on a heparin drip. Due to continued abdominal pain, CTA with runoff was obtained and revealed a large abdominal aortic clot with extension into the SMA, clot of the bilateral femoral arteries, bilateral renal infarcts, splenic infarcts, as well as a right retroperitoneal hematoma. At this point, given the futility of further treatment, the goals of care discussion was held with the patient, and family and she chose to become DNR/DNI and passed away the following day.

## Discussion

The vasoactive effects of cocaine are well documented and easily detected with routine imaging such as TTE and cardiac catheterization. These notably affect the cardiovascular and cerebrovascular systems, with serious consequences on the morbidity and mortality of patients. Well-reported cocaine-related complications include sudden cardiac death, acute myocarditis, dilated cardiomyopathy, life-threatening arrhythmias, myocardial infarction, and dissections of the coronary, aortic, and carotid arteries, to name a few [4,5]. However, less common are those that affect the gastrointestinal system; among them are ischemic ulcerations of the stomach and duodenum, segmental colitis, and pancolitis [6]. Cocaine abuse can also cause mesenteric ischemia and gangrene, which may result in small and large bowel perforation leading to intraperitoneal hemorrhage [7].

Our patient was also noted to have gangrenous changes intraoperatively. This is even more uncommon in a patient being treated for acute coronary syndrome (ACS) and left ventricle (LV) thrombus. The temporality of these complications occurring together has been largely undocumented. Although there is a possibility this may be an embolic disease process, it would be less likely given the use of a heparin drip after the identification of this patient’s LV thrombus.

The complex pathophysiology of cocaine use leading to vascular damage can be summarized as follows: cocaine ingestion causes acute stress and leads to endothelial damage promoting an increase in fibrinogen and von Willebrand factor leading to platelet activation and aggregation and ultimately the formation of blood clots [8]. Additionally, inflammation and atherosclerosis are potentially lethal vascular effects of cocaine use that have acute and chronic systemic impact. Cocaine also creates an elevated immune system inflammatory state with decreased basal anti-inflammatory markers (e.g., interleukin-10) and increased pro-inflammatory cytokines (e.g., tumor necrosis factor-alpha, Interleukin 1β) all contributing to vascular disease (e.g., endocarditis) [9]. Regarding the pathophysiology of cocaine use and vascular damage along with clot formation, it is evident that in the absence of other risk factors, our patient most likely was affected by widespread thrombi to multiple areas of the body (LV thrombus, SMA thrombus, renal infarct, splenic infarct, and vasospasm leading to an MI). The likelihood of all these events happening together is quite low as evidenced by the lack of literature on multi-organ thrombosis secondary to cocaine use.

Our patient presented a therapeutic dilemma in the setting of a coexisting retroperitoneal hematoma that developed following heparin drip administration, preventing therapeutic anticoagulation despite multiple systemic thrombi. It is important to recognize the delirious effects of cocaine use while treating patients on a day-to-day basis. The rise of cocaine abuse in the young population entails a very important diagnostic problem regarding uncommon diseases in this age group, such as acute small bowel ischemia [10]. In most cases, the related morbidity and mortality are time-dependent, and a high index of suspicion is therefore required to establish a prompt and proper diagnosis.

## Conclusions

Considering the prevalence of cocaine use and its widespread complications, more studies should be aimed at the management of multi-organ thrombosis, like that in our patient, to determine the benefit of immediate surgical intervention versus oral or intravenous anticoagulation.

Furthermore, the relevance of previous MI and the implications of discontinuing antiplatelet therapy concurrent with cocaine use is essential for optimizing the management and outcomes of patients with cardiovascular disease who engage in substance abuse. This underscores the need for comprehensive risk assessment, multidisciplinary collaboration, and patient education to mitigate the potential adverse effects of cocaine on the cardiovascular system. The symptoms and manifestations of cocaine abuse may occur simultaneously from multiple systems. Concurrently, with the potential of illicit drugs, such as cocaine, to cause intravascular thrombosis across the body, it is imperative for physicians to be cautious of any such symptoms.
